# Association of an *ACSL1 *gene variant with polyunsaturated fatty acids in bovine skeletal muscle

**DOI:** 10.1186/1471-2156-12-96

**Published:** 2011-11-11

**Authors:** Philipp Widmann, Karin Nuernberg, Christa Kuehn, Rosemarie Weikard

**Affiliations:** 1 Research Unit Molecular Biology, Leibniz Institute for Farm Animal Biology (FBN), Wilhelm-Stahl-Allee 2, Dummerstorf, 18196, Germany; 2 Research Unit Muscle Biology and Growth, Leibniz Institute for Farm Animal Biology (FBN), Wilhelm-Stahl-Allee 2, Dummerstorf, 18196, Germany

## Abstract

**Background:**

The intramuscular fat deposition and the fatty acid profiles of beef affect meat quality. High proportions of unsaturated fatty acids are related to beef flavor and are beneficial for the nutritional value of meat. Moreover, a variety of clinical and epidemiologic studies showed that particularly long-chain omega-3 fatty acids from animal sources have a positive impact on human health and disease.

**Results:**

To screen for genetic factors affecting fatty acid profiles in beef, we initially performed a microsatellite-based genome scan in a F_2 _Charolais × German Holstein resource population and identified a quantitative trait locus (QTL) for fatty acid composition in a region on bovine chromosome 27 where previously QTL affecting marbling score had been detected in beef cattle populations. The *long-chain acyl-CoA synthetase 1 (ACSL1) *gene was identified as the most plausible functional and positional candidate gene in the QTL interval due to its direct impact on fatty acid metabolism and its position in the QTL interval. ACSL1 is necessary for synthesis of long-chain acyl-CoA esters, fatty acid degradation and phospholipid remodeling. We validated the genomic annotation of the bovine *ACSL1 *gene by *in silico *comparative sequence analysis and experimental verification. Re-sequencing of the complete coding, exon-flanking intronic sequences, 3' untranslated region (3'UTR) and partial promoter region of the *ACSL1 *gene revealed three synonymous mutations in exons 6, 7, and 20, six noncoding intronic gene variants, six polymorphisms in the promoter region, and four variants in the 3' UTR region. The association analysis identified the gene variant in intron 5 of the *ACSL1 *gene (*c.481-233A>G*) to be significantly associated with the relative content of distinct fractions and ratios of fatty acids (e.g., n-3 fatty acids, polyunsaturated, n-3 long-chain polyunsaturated fatty acids, trans vaccenic acid) in skeletal muscle. A tentative association of the *ACSL1 *gene variant with intramuscular fat content indicated that an indirect effect on fatty acid composition via modulation of total fat content of skeletal muscle cannot be excluded.

**Conclusions:**

The initial QTL analysis suggested the *ACSL1 *gene as a positional and functional candidate gene for fatty acid composition in bovine skeletal muscle. The findings of subsequent association analyses indicate that *ACSL1 *or a separate gene in close proximity might play a functional role in mediating the lipid composition of beef.

## Background

In recent decades, the continuing accumulation of knowledge and the increasing number of reports providing evidence regarding the beneficial health effects of polyunsaturated fatty acids (PUFA) have attracted the attention of the medical and public community. Consumers are becoming increasingly aware of the relationships between diet and health and also of the importance of the diet for general physical and mental wellbeing [1,2]. Many clinical and epidemiologic studies have indicated a positive impact of long-chain omega-3 fatty acids (n-3 long-chain polyunsaturated fatty acids, n-3 LC-PUFA) on human health and disease. Beneficial effects of n-3 LC-PUFA are described in infant development, cancer, and cardiovascular diseases (e.g., [3-6]), lipid and glucose metabolism (e.g., [7-10]), inflammation (e.g., [11,12]), and more recently, in various mental illnesses including depression, attention-deficit hyperactivity disorder, and dementia (e.g.,[13]). It has been demonstrated that diets containing higher levels of n-3 LC-PUFA [namely DHA (docosahexaenoic acid; C22:6n-3) and EPA (eicosapentaenoic acid; C20:5n-3)], may reduce cardiovascular risk in diabetes by inhibiting platelet aggregation, improving lipid profiles, and reducing cardiovascular mortality. Thus, n-3 LC-PUFA were particularly recommended to people with diabetes and metabolic disorders associated to obesity [5,14]. Their beneficial health effects may be mediated through multiple distinct mechanisms, including alterations in cell membrane composition and function, gene expression, or eicosanoid biosynthesis [15,16]. It is known that n-3 LC-PUFA can exert important metabolic effects due to their ability to modulate the transcription of regulatory genes with function in lipid metabolism [17-21].

The n-3 LC-PUFA, like DHA and EPA, are particularly abundant in oily cold-water fish and seafood, however, they are also present in other animal products (e.g., ruminant meat and milk) but in lower concentrations. Increases of n-3 LC-PUFA content in the human diet can be achieved by dietary supplementation, but there is also a potential to alter the natural fatty acid (FA) profile in food from animals. FA composition of meat and milk reflects both, FA biosynthesis in the respective animal tissue and FA composition of ingested nutrients. A recent study showed that cattle and lambs fed grass-diet in the period before slaughter had an increased content of beneficial FAs in meat, and that subsequent moderate consumption of the respective meat had resulted in increased plasma and platelet n-3 LC-PUFA concentrations in healthy human individuals [22]. A ruminant diet on grass, which is rich in α-linolenic acid (C18:3n-3, ALA) compared to cereal-based concentrate diet can influence the FA profile of meat in the desired direction and improve its nutritional value [23-25]. However, the link between nutritional intake of FAs and its subsequent concentration in skeletal muscle is stronger in monogastric animals (pigs, poultry) than in ruminants due to hydrogenation of dietary FAs in the rumen (e.g., [26]).

In addition to the environmental conditions, genetic factors may also have a substantial effect on the variability of FA composition in animal products, especially for ruminants [27]. Consequently, genetic selection and breeding of animals with favorably enriched n-3 LC-PUFA content in skeletal muscle can provide a rich source of the desired beneficial FAs for the human diet. Therefore, it is necessary to elucidate the molecular-genetic background of fatty acid composition in bovine skeletal muscle for identifying the genes or gene variants favorable for human nutrition.

Numerous quantitative trait loci (QTL) affecting meat quality traits in cattle like marbling and FA composition have been identified on a variety of bovine chromosomes (http://www.animalgenome.org/cgi-bin/gbrowse/bovine/), which enabled subsequent identification of positional candidate genes, which are located in the vicinity of identified QTL and have putative physiological functions regarding FA synthesis in skeletal muscle. These candidate genes for lipid-associated traits have been studied for their possible role regarding phenotypic variation observed between and within breeds. DNA variants in a variety of genes involved in lipid synthesis and FA metabolism have been found to influence FA composition in bovine muscle tissue and carcass (*SCD1*, [28-34], *SREBP-1 *[29], *FASN *[29,34-37], *FABP4 *and *LXRα *[38], *GH *[29], *ACACA *[39], myostatin [40,41], leptin [33]).

However, the biochemical processes and the molecular background affecting the genetic variability of the complex polygenic trait of FA composition are not yet completely understood, particularly with regard to European cattle breeds, because the majority of recent studies have been performed on the very specific genetic background of Japanese Black cattle.

Therefore, the aim of this study was to identify genetic factors affecting the variation of FA composition in bovine skeletal muscle. For our study, we took advantage of a unique F_2 _resource population generated from the major European cattle breeds Charolais and German Holstein by means of embryo transfer and foster mothers [42]. In previous studies, this population had been shown to segregate for two major loci (*NCAPG *and *MSTN*) associated with prenatal and pubertal growth, postnatal body composition and general lipid deposition [43,44].

## Results and discussion

The animals from our resource cross population were kept and fed at standardized uniform conditions and slaughtered at the same age. Therefore, we can exclude exogenous factors due to differences in herd, age, feeding and gender. Consequently, differences in skeletal muscle fatness or FA composition should be due to differences in endogenous factors of the animals like the genetic background. The primary focus of our study was to discover phenotypic differences of FA composition in skeletal muscle between the individual animals of the resource population due to genetic variation.

### QTL analysis and identification of ACSL1 as a positional and functional candidate gene

**Figure 1:**
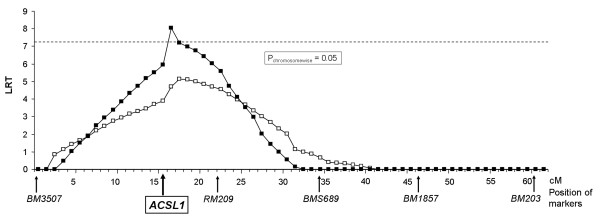
**QTL for the relative content of n-3 long chain PUFA (n-3 LC PUFA) on BTA27**. Solid boxes: *ACSL1 *c.481-233A>G effect not included in the model, open boxes: *ACSL1 *c.481-233A>G included in the model as a fixed effect. LRT significance threshold is indicated by a dashed line (α = 0.05: 7.23).

An initial QTL analysis in the Charolais × German Holstein cross population identified a QTL for FA composition on bovine chromosome 27 (BTA27) as exemplified for n-3 LC-PUFA in Figure 1. In our study, the trait n-3 LC-PUFA represents n-3 PUFA exceeding a carbon chain length of C18. The QTL interval corresponded to a region, where previously QTL affecting marbling had been detected in a Bos indicus × Bos taurus cross and two commercial US Angus populations [45,46]. The QTL explained 20.5% variance in the model calculated as the relative reduction of the residual variance due to including the QTL in the model [47].

Furthermore, QTL for FA composition, myristic acid, (C14:0) and oleic acid (C18:1) content, have been reported in this chromosomal region in a Jersey × Limousin back-cross cattle population [48]. In our study, the QTL interval affecting FA composition in skeletal muscle displayed a peak between 15 and 16 cM on our genetic map of BTA27 corresponding to a genomic position at approximately 16 Mb on the current bovine genome assembly of the chromosome (NCBI mapviewer, build 5.2, http://www.ncbi.nlm.nih.gov/projects/mapview/map_search.cgi?taxid = 9913).

Based on its chromosomal position and integration in biochemical pathways of lipid metabolism, we identified the *acyl-CoA synthetase long-chain family member 1 *(*ACSL1*) gene as the most plausible positional and functional candidate gene underlying the QTL with effect on FA composition on BTA27. The *ACSL1 *gene is located exactly under the peak of the QTL interval. Its protein, the ACSL1 enzyme, is known to catalyze the first step of activation of long-chain (LC) FAs by converting them into LC acyl CoA thioesters for channeling towards chain elongation, triacylglyceride synthesis or FA oxidation [49]. ACSL1 has a key function in both the synthesis of cellular lipids and FA degradation, and is also necessary for phospholipid remodeling [50]. Due to its physiological biochemical function, it can be suggested that ACSL1 plays an important role in lipid metabolism, insulin resistance and obesity. Recently, a study in humans reported that a gene variant located in intron 1 of the *ACSL1 *gene can influence the metabolic syndrome risk (characterized by insulin resistance, dyslipidaemia, abdominal obesity and hypertension associated to type 2 diabetes), and that this *ACSL1 *genotype-dependent effect can be modulated by dietary PUFA intake suggesting a gene-nutrient interaction [51].

### Structure analysis and screening for polymorphisms of the ACSL1 gene

**Figure 2:**
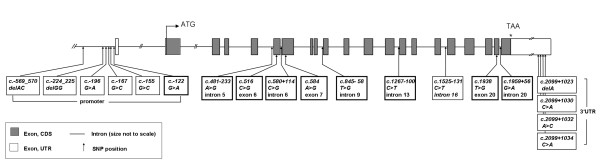
**Structure of the bovine *ACSL1 *gene and detection of polymorphic sites**. Reference mRNA sequence: NM_001076085.1. SNPs included in the association analysis are bold framed.

Although sequences for the *ACSL1 *gene and protein were deposited in the bovine genome databases, we found inconsistencies regarding the structural annotation of the gene in the bovine genome assemblies. A correct and conclusive structural gene annotation is a prerequisite for subsequent screening for gene variants and analysis of their functional relevance. Therefore, the first step of our study focused on the experimental confirmation of the structure of the *ACSL1 *gene on the genomic and cDNA level. Experimental verification by RT-PCR, re-sequencing and comparative sequence analyses confirmed the genomic annotation of the bovine *ACSL1 *gene in the alternate UMD_3.1 genome assembly (Figure 2), which is in contrast to the reference genome assembly Btau4.2 (http://www.ncbi.nlm.nih.gov/projects/mapview/map_search.cgi?taxid = 9913).

Re-sequencing of DNA from pools and individuals differing in IMF content and Δ^9 ^desaturase activity index included a total of 8.5 Kb of genomic DNA. Comparative sequence analysis revealed a total of 19 single nucleotide polymorphisms (SNPs) in the targeted gene regions (Table 1). Three synonymous exonic (exons 6, 7 and 20), six intronic (introns 5, 6, 9, 13, 16 and 20), six SNPs in the promoter region and four SNPs in the 3'UTR of the bovine *ACSL1 *gene were detected (Figure 2, Table 1). Eleven out of these SNPs identified in our study were novel and not previously represented in the SNP database (version 133) at NCBI.

**Table 1:** Identified SNPs within the ACSL1 locus and positions on the bovine genome assemblies

SNP ID relative to coding sequence*	Gene region	Variation relative to reference sequence	Position on NW_001494406.2 (Btau4.2)	Position on NW_003104605.1 (UMD_3.1)	Allele frequency	SNP accession number (dbSNP, NCBI ss#)
c.-569_570del AC	Promoter	Indel TG	1918795	389858	Not analyzed	ss469271165
c.-224_225del GG	Promoter	Indel (C)_5-7_	1918451	389615	Not analyzed	ss469271166
c.-196G>A	Promoter	C>T	1918422	3894586	Not analyzed	ss469271167
c.-167G>C	Promoter	C>G	1918393	3894557	Not analyzed	ss469271168
c.-151G>C	Promoter	C>G	1918377	3894541	Not analyzed	ss469271169
**c.-122G>A**	Promoter	C>T	1918348	3894512	0.67 (G)/0.33 (A)	ss469271170
**c.481-233A>G**	Intron 5	T>C	1876389	3852106	0.73 (A)/0.27 (G)	ss469271171
**c.516C>G**	Exon 6	G>C	1876121	3852284	0.57 (C)/0.43 (G)	ss469271172
**c.580+114C>G**	Intron 6	G>C	1875943	3852552	0.33 (C)/0.67 (G)	ss469271173
**c.584A>G**	Exon 7	T>C	1875838	3852001	0.07 (G)/0.94 (A)	ss469271174
**c.845-58T>G**	Intron 9	A>C	1871919	3848082	0.75 (T)/0.21 (G)5	ss469271175
**c.1267-100C>T**	Intron 13	G>A	1864210	3840373	0.24 (T)/0.76 (C)	ss469271176
c.1525-131C>T	Intron 16	G>A	1859370	3835533	Not analyzed	ss469271177
**c.1938T>G**	Exon 20	A>C	Not annotated	3831586	0.75 (T)/0.25 (G)	ss469271178
**c.1959+56G>A**	Intron 20	C>T	Not annotated	3831509	0.24 (A)/0.76 (G)	ss469271179
c.2099+1023del A	3'UTR	Indel A	1853870	3829944	Not analyzed	ss469271180
c.2099+1030 C>A	3'UTR	C>A	1853863	3829937	Not analyzed	ss469271181
c.2099+1032 A>C	3'UTR	A>C	1853861	3829935	Not analyzed	ss469271182
c.2099+1034 C>A	3'UTR	C>A	1853859	3829933	Not analyzed	ss469271183

*SNP nomenclature according to the translation start codon ATG, reference sequence NM_001076085.1. SNPs marked in bold were included in the association analysis. Genomic positions of the SNPs were inferred from the current versions of the refererence and alternative bovine genome assemblies Btau4.2 and UMD_3.1 available at NCBI (http://www.ncbi.nlm.nih.gov/genome/guide/cow/index.html).

### Association of ACSL1 gene variants with PUFA profile in skeletal muscle

The association analysis included all exonic and intronic *ACSL1 *gene variants (except for the one in intron 16) and one SNP in the promoter region, which were identified by re-sequencing and validated by genotyping in the Charolais × German Holstein resource population. The nine SNPs analyzed in the Holstein × Charolais cross bred population showed a minor allele frequency ≥ 0.2 in the analyzed data set (Table 1). Intragenic linkage disequilibrium (LD) analysis revealed a strong LD between the SNPs in intron 20, exon 20, intron 13 and intron 9 (r^2 ^>0.9), whereas there was only a moderate LD (0.5 < r^2 ^<0.6) between these SNPs and the one in intron 5.

The association analysis with intragenic *ACSL1 *SNPs revealed that the SNP located in intron 5 of the *ACSL1 *gene (*c.481-233A>G*) showed the most significant associations with FA composition in skeletal muscle. The gene variant *ACSL1 c.481-233A>G *was significantly associated with the relative content of distinct fractions of unsaturated FAs, n-3 FA, PUFA, n-3 LC-PUFA and docosapentaenoic fatty acid (DPA) as well as with the absolute content of total FA, MUFA, and trans vaccenic acid (C18:1trans-11) in *M. longissimus dorsi *(Table 2). The results revealed that the *c.481-233A *allele of this gene variant is strongly associated with a higher relative level of n-3 FA, PUFA, DPA, and n-3 LC-PUFA. In contrast, the c.481-233A allele showed a decreasing effect on content of C18:1trans-11, total FA, and MUFA, and tended to be associated with a lower IMF content in skeletal muscle compared to the *c.481-233G *allele. The *c.481-233A *allele had a higher freqeuncy (73%) in the analyzed population compared to the alternative allele (27%).

**Table 2:** Association of the SNP in intron 5 of the *ACSL1 *gene (*c.481-233A>G*) with variation in intramuscular fatty acid composition and fat content

	Model without IMF as covariate							Model with IMF as covariate					
Trait*	LRT	p-value	Effect allele A	SE	Effect allele G	SE	Var [%]**	LRT	p-value	Effect allele A	SE	Effect allele G	SE
Total FA [mg]*^&^	6.2	0.0130	3.70	0.07	3.88	0.07		3.8	0.0507				
MUFA [mg]*^&^	5.9	0.0154	3.29	0.08	3.48	0.09		2.9	0.0874				
n-3 FA [%]*^#^	9.7	0.0018^a^	0.16	0.05	0.01	0.06	11.4	5.8	0.0159	0.42	0.04	0.36	0.04
PUFA [%]*^#^	7.1	0.0079^b^	1.12	0.05	0.98	0.06	3.5	3.9	0.0477				
n-3 LC PUFA [%]^#^	6.7	0.0099^b^	0.24	0.03	0.16	0.04	3.4	3.5	0.0628				
C18:1trans-11 [mg]*^&^	10.5	0.0012^a^	1.31	0.10	1.63	0.12	6.5	8.1	0.0045^a^	0.86	0.09	1.07	0.10
C22:5n-3 [%]^#^	7.2	0.0071 ^b^	0.16	0.02	0.11	0.03	3.6	5.9	0.0150	0.27	0.02	0.23	0.02
PUFA/SFA*	7.3	0.0069^b^	-0.79	0.06	-0.94	0.07	3.6	4.2	0.0407				
P/S*	6.7	0.0099^b^	-1.02	0.05	-1.15	0.06	3.2	3.2	0.0758				
LA/ALA*	4.9	0.0265	1.03	0.03	0.97	0.03		1.9	0.1720				
IMF [%]	3.1	0.0776	1.11	0.18	1.42	0.21	1.7						

*Trait data log-transformed, ^&^absolute content of fatty acids (mg/100 g of skeletal muscle), ^#^relative content of fatty acids (percentage of the respective fatty acid fraction relative to total fatty acid amount)

LTR: likelihood ratio test, p: significancy of allelic effects, Var: variance explained, SE: standard error, ^a^q-value <0.05, ^b^q-value <0.1, **% variance in the model calculated as the relative reduction of the residual variance due to including the SNP in the model [47].

Allelic effects of the *ACSL1 *SNP (c.481-233A>G) on different FA fractions: n-3 fatty acids (n-3 FA = C18:3n-3 + C18:4n-3 + C20:5n-3 + C22:5n-3 + C22:6n-3), poly-unsaturated fatty acids (PUFA = n-3 FA + n-6 FA [C18:2n-6 + C18:3n-6 + C20:2n-6 + C20:3n-6 + C20:4n-6 + C22:2n-6 + C22:4n-6]), n-3 long-chain PUFA (n-3 LC-PUFA = C20:3n-3 + C22:6n-3 + C22:5n-3 + C20:5n-3), total FA, mono-unsaturated fatty acids (MUFA), docosapentaenoic fatty acid (DPA, C22:5n-3), trans vaccenic acid (TVA, C18:1trans-11) and the ratios: PUFA/SFA (saturated fatty acids = C12:0 + C14:0 + C16:0 + C17:0 + C18:0 + C20:0 + C24:0), P/S (C18:2 n-6 + C18:3 n-3/C14:0 + C16:0 + C18:0), LA/ALA (C18:2 n-6/C18:3 n-3), and the intramuscular fat content (IMF).

Although the *c.481-233A *allele tends to be associated with a slightly lower total IMF content, the relative content of the FA fractions, n-3 FA, PUFA, DPA, and n-3 LC-PUFA, known to exert health-beneficial effects in humans is highly increased indicating a higher nutritional value for beef originating from animals with the favorable *ACSL1 *allele.

The strongest allelic effect of the *ACSL1 c.481-233A>G *locus was observed for n-3 FA content. This trait also includes the polyunsaturated C18 fatty acids, α-linolenic acid (ALA, C18:3n-3) and stearidonic acid (C18:4n-3). The n-3-FA content is different to the trait n-3 LC-PUFA, which exclusively comprises n-3 FA with a chain length > C18. As an essential FA, ALA cannot be synthesized by mammalian species and must be obtained from the diet. The ALA concentration in skeletal muscle, therefore, could be linked to the dietary absorption. However, the standardized concentrate-based feeding regimen in our study provides uniform feeding conditions for the animals. ALA is the precursor for the n-3 FA pathway [52] by serving as parent FA for the synthesis of stearidonic acid and n-3 LC-PUFA (EPA, DPA, and DHA) via sequential steps of desaturation and/or chain-elongation. The association of *ACSL1 c.481-233A>G *with DPA and with n-3 LC-PUFA (containing n-3 FA exceeding a chain length of C18) could suggest that a substantial proportion of their precursor ALA might be activated and channeled to chain elongation processes.

The trait PUFA comprises both FA types, the n-6 and n-3 FA. The *ACSL1 c.481-233A>G *variant showed no significant impact on n-6 FA content and thus, its association with PUFA could be due to its effect on the trait's component n-3 FA.

Interestingly, the *ACSL1 *gene variant *c.481-233A>G *that affected FA profiles in bovine skeletal muscle had no significant influence on the ratio n-6/n-3 FA in this tissue. Considering the standardized uniform feeding regimen in our study, this result could support the findings from other studies, which indicate that the n-6/n-3 FA ratio may be affected more by feeding than by genetics [53,54]. In contrast, we found the *ACSL1 *gene variant *c.481-233A>G *to be associated with the LA/ALA (C18:2 n-6/C18:3 n-3) ratio. Furthermore, we observed significant associations of this gene variant with the ratios PUFA/SFA and P/S in our study, both representing characteristics of meat quality and widely used to evaluate the nutritional value of meat fat content. Again, the c.481-233A allele revealed an increasing effect on these ratios compared to the *c.481-233G *allele.

In contrast to the increasing effect associated with the *c.481-233A *allele on the relative content of the FA fractions, n-3 FA, PUFA, DPA, and n-3 LC-PUFA, and the PUFA/SFA and P/S ratios, we observed a decreasing effect of this allele on the absolute content of the trans vaccenic acid C18:1*trans-*11 in skeletal muscle in our study. This effect was in concert with the associated parallel decrease in total FA and MUFA content in the tissue. The effect on C18:1trans-11 is of particular interest, because trans vaccenic acid is a precursor of conjugated linoleic acid (CLAcis-9, trans-11) generation. CLAs are believed to have several important physiological functions, including anti-carcinogenic, anti-atherogenic, immunomodulating, growth and lean body mass promoting effects [55]. Thus, targeted selection of cattle carrying the homozygous *c.481-233A/c.481-233A *genotype in the *ACSL1 *gene would possibly be accompanied by detrimental effects on the CLA profile in skeletal muscle.

There is the open question, whether the significant effects of the *ACSL1 *gene variant *c.481-233A>G *on FA composition were due to general fatness differences in skeletal muscle, which is supported by several QTL for marbling in the targeted chromosomal region, or whether the effects were associated with the *ACSL1 *gene variant *c.481-233A>G*. Alternatively, the effects of this gene variant might modulate the accumulation of specific FAs in skeletal muscle. To address this issue, we extended our association analysis and fitted IMF as a covariate in the model. When adjusting for IMF (Table 2), the association of the *ACSL1 *gene variant *c.481-233A>G *with absolute content of trans vaccenic acid in skeletal muscle remained significant, whereas the other associations dropped below a stringent threshold of statistical significance (Bonferroni q < 0.1) and were only tentatively significant (e.g., for relative content of n-3 FA and DPA). Thus, we cannot exclude that variants in the bovine *ACSL1 *gene may exert a substantial effect on total intramuscular fat content, which indirectly affects intramuscular composition of specific FA fractions. However, as the results for trans vaccenic acid demonstrate, it is suggested that there are also direct effects associated with the *ACSL1 *gene variant *c.481-233A>G *on intramuscular content of specific FAs.

## Conclusions

Due to our observation that the *c.481-233A>G *SNP in intron 5 of the *ACSL1 *gene cannot fully explain the QTL variance (Figure 1), we conclude that this gene variant is presumably not causal, but in LD to another not yet detected polymorphism in its close vicinity affecting FA composition in bovine skeletal muscle. Presumably, these effects are not exclusively the consequence of variation in intramuscular fat content, but due to effects on specific FA. Prior to selective breeding of cattle carrying the desired genotype of the *ACSL1 *gene variant *c.481-233A>G *in order to produce meat with specific FA profiles, the association between *c.481-233A>G *and FA composition has to be confirmed in the particular target cattle population.

Nevertheless, our results indicate that the *ACSL1 *gene might play a functional role in mediating the FA composition in bovine skeletal muscle and provide a basis to further elucidate the function of the *ACSL1 *gene and its coordinated network with genes integrated in FA metabolism to dissect the molecular background of lipid composition of beef.

## Methods

### Animals and phenotypes

The generation of the Charolais × German Holstein resource cross population (SEGFAM), details regarding feeding and housing of the animals analyzed in our study, have been previously described [43,44]. The animals were kept under standardized environmental and feeding conditions in barn facilities at the Leibniz Institute for Farm Animal Biology (FBN). After birth, the calves were fed a milk/replacer/hay/concentrate diet *ad libitum *until day 121. Thereafter, the animals received a feed ration of concentrates and chaffed hay with a hay to concentrate ratio of 1:3 and an energy content of 12.7 MJ ME/kg dry matter fed *ad libitum *until slaughter. The animals were kept in a tight stall barn with individual daily feed recording. At the age of 18 months (547 days of age), the male animals were slaughtered, and a detailed dissection of the carcass was performed. A wide range of phenotypic data related to beef production and beef quality including FA composition were recorded including FA composition of selected skeletal muscles.

Analysis of FA composition of lipids involving 26 different FAs in skeletal muscle (*M. longissimus dorsi*) was determined for 156 F_2 _bulls using capillary gas chromatography as described previously [56]. The absolute amount of FAs in skeletal muscle was determined from 2 g of skeletal muscle and calculated as mg/100 g tissue. The relative content of individual fatty acids was calculated as percentage of the total amount of FAs extracted. Based on the data obtained for individual fatty acids, sums of specific fatty acid fractions were calculated: saturated fatty acids (SFA), unsaturated fatty acids (UFA), monounsaturated fatty acids (MUFA), trans fatty acids (TFA), n-3 fatty acids (n-3 FA), n-6 fatty acids (n-6 FA), polyunsaturated fatty acids (PUFA) and n-3 long-chain PUFA (n-3 LC-PUFA). Furthermore, the ratios n-6/n-3 FA, MUFA/SFA, PUFA/SFA, P/S and LA/ALA as well as four different Δ^9 ^desaturase indices [57] were calculated. Intramuscular fat (IMF) content (percentage in 100 g tissue) was ascertained in *M. longissimus dorsi *by FoodScan Lab (FOSS) as described previously [56]. The phenotypic traits for FA composition of IMF included in our study are summarized in Table 3.

**Table 3:** Phenotypic traits characterizing fatty acid composition in skeletal muscle

Trivial name	Abbreviation	Mean of absolute content (mg/100 g)	SD	Mean of relative content (%)	SD
Lauric acid	C12:0	2.83*	1.80	0.10	0.04
Myristoleic acid	C14:1cis-9	18.53	17.85	0.62	0.31
Myristic acid	C14:0	92.12*	65.50	3.10*	0.66
Palmitoleic acid	C16:1cis-9	109.33*	76.59	3.73	0.83
Palmitic acid	C16:0	823.94*	490.88	28.77	1.86
Heptadecenoic acid	C17:1cis-10	23.48*	12.51	0.85	0.18
Margaric acid	C17:0	34.94*	18.13	1.26*	0.25
Stearic acid	C18:0	373.40*	199.95	4.96*	1.80
Oleic acid	C18:1cis-9	981.37*	578.52	34.35	2.60
Vaccenic acid	C18:1cis-11	43.60*	30.60	13.41	1.65
trans Vaccenic acid	C18:1trans-11	28.40*	20.18	1.52*	0.33
Linoleic acid (LA)	C18:2n-6	121.45*	29.94	0.99*	0.43
Linolelaidic acid	C18:2trans-9, trans-12	4.11*	3.39	0.11	0.13
Conjugated linoleic acid	CLAcis-9, trans-11	5.99*	4.73	0.20	0.09
α-Linolenic acid (ALA)	C18:3n-3	12.99	4.97	0.50*	0.14
Stearidonic acid (SDA)	C18:4n-3	2.70	3.36	0.15	0.10
Arachidic acid	C20:0	2.94	1.90	0.11	0.06
Eisosenoic acid	C20:1n-9	4.21*	2.80	0.35	0.15
Eisosatrienoic acid (ETE)	C20:3n-3	8.15*	1.86	0.11*	0.05
Arachidonic acid (AA)	C20:4n-6	33.89	7.60	1.48*	0.74
Timnodonic acid, EPA	C20:5n-3	2.41*	0.70	0.14*	0.03
Erucic acid	C22:1n-9	0.46*	0.24	0.02*	0.01
Adrenic acid	C22:4n-6	6.20	1.53	0.28	0.13
Clupadonic acid, DPA	C22:5n-3	6.51*	1.34	0.26*	0.11
Cervonic acid, DHA	C22:6n-3	0.92	0.45	0.04	0.03
Lignoceric acid	C24:0	0.82	0.44	0.03*	0.02
∑ Saturated fatty acids	SFA	1352.06*	777.93	47.55	2.38
∑ Unsaturated fatty acids	UFA	1463.62*	777.61	52.45	2.38
∑ Polyunsaturated fatty acids	PUFA	208.34*	44.62	8.57*	3.02
∑ Monounsaturated fatty acids	MUFA	1208.85*	726.67	42.19*	3.16
∑ trans fatty acids	TFA	43.92*	24.70	1.58	0.47
∑ n-3 fatty acids	n-3FA	24.75	6.42	1.01*	0.35
∑ n-3 long-chain PUFA	n-3 LCPUFA	9.58	2.21	0.42*	0.20
∑ n-6 fatty acids	n-6FA	167.28*	34.88	6.94	2.64
∑ n-6 long-chain PUFA	n-6 LCPUFA	40.09	8.62	1.78	0.85
∑ Total fatty acids	FA	2743.81*	1524.08		
Ratio n-6/n-3	n-6/n-3	6.96	1.42		
Ratio MUFA/SFA	MUFA/SFA	0.89	0.09		
Ratio PUFA/SFA	PUFA/SFA	0.18*	0.07		
Ratio P/S	P/S	0.12*	0.05		
Ratio C18:2n-6/C18:3n-3	LA/ALA	9.86*	2.51		
Δ^9^-desaturase index MUFA	Δ9MUFA	46.39	2.56		
Δ^9^-desaturase index C14	Δ9C14	16.31	6.65		
Δ^9^-desaturase index C16	Δ9C16	11.40	2.08		
Δ^9^-desaturase index C18	Δ9C18	72.16	3.34		
Intramuscular fat content	IMF			2.56	1.13

SD: standard deviation

*Data displaying distributions significantly different from normality (p < 0.01) were log-transformed.

SFA = C12:0 + C14:0 + C16:0 + C17:0 + C18:0 + C20:0 + C24:0

MUFA = C14:1 + C16:1 + C17:1 + C18:1 + C20:1 + C22:1 + C18:1cis-9 + C18:1cis-11 + C18:1trans-11

UFA = MUFA + PUFA

Total FA = sum of all fatty acids determined

n-3FA = C18:3n-3 + C18:4n-3 + C20:3n-3 + C20:5n-3 + C22:5n-3 + C22:6n-3

n-6FA = C18:2n-6 + C18:3n-6 + C20:2n-6 + C20:3n-6 + C20:4n-6 + C22:2n-6 + C22:4n-6

PUFA = n-3FA + n-6FA

n-3 LC-PUFA = C20:3n-3 + C22:6n-3 + C22:5n-3 + C20:5n-3

TFA = C18:1trans-11 + C18:2trans-9, trans-12

LA/ALA = C18:2n-6/C18:3n-3

P/S = C18:2 n-6 + C18:3 n-3/C14:0 + C16:0 + C18:0

Δ^9^MUFA = [(C14:1 + C16:1 + C17:1 + C18:1cis-9 + CLAcis-9, trans-11)/(C14:1 + C16:1 + C17:1 + C18:1cis-9 +

C18:1trans-11 + C14:0 + C16:0 + C17:0 + C18:0 + CLAcis-9, trans-11)] × 100

Δ^9^C14 = [C14:1/(C14:0 + C14:1)] × 100

Δ^9^C16 = [C16:1/(C16:0 + C16:1)] × 100

Δ^9^C18 = [(C18:1cis-9 + CLAcis-9, trans-11/(C18:0 + C18:1cis-9 + C18:1trans-11 + CLAcis-9, trans-11)] × 100

### QTL analysis

An initial QTL scan comprising 244 microsatellite markers [58] for variation of FA composition in skeletal muscle had pinpointed a region on bovine chromosome 27 (BTA27) with effect on n-3 PUFA content in skeletal muscle. Five microsatellite markers located on BTA27 (*BM3507, RM209, BMS689, BM1857, BM203*) had been genotyped in all 733 P_0_, F_1_, and F_2 _individuals of the Charolais × German Holstein resource population.

The respective QTL interval pointed to a chromosomal region on BTA27 harboring the *acyl-CoA synthetase long-chain family member 1 *(*ACSL1*) gene according to the sequence assembly of the chromosome. Therefore, in a second step of our analysis, nine intragenic *ACSL1 *SNPs (Figure 1) were added to the initial marker set. All microsatellite markers and all genotyped *ACSL1 *SNPs were included to calculate a genetic map using CRIMAP Version 2.50 [59], incorporating modifications by Ian Evans and Jill Maddox (University of Melbourne). The resulting genetic map was applied in the QTL analyses with a variance component QTL model as implemented in Qxpak [60] and essentially as described previously [43]:

y=Fb+Zu+Qg+e;

where y is a vector of phenotypes, *b *is a vector of the fixed effects (slaughter year, *NCAPG I442M *genotype), *u *is the vector of individual infinitesimal polygenic effects, *g *is a vector of the additive QTL effects not fixed within founder breeds; **F**, **Z **and **Q **represent the incidence matrices for the fixed, polygenic and the QTL effect, respectively, and *e *is the vector of random residuals. An MCMC algorithm was used to calculate identity-by-descent probabilities as implemented in Qxpak. The *NCAPG I442M *mutation was included in the model, because previous analyses had shown a major effect of the mutation on carcass lipid deposition and growth in the resource population [44].

Statistical significance of the QTL analyses was tested by a likelihood-ratio test (LRT). Significance thresholds for the LRT were determined according to [61], considering one chromosome with a length of 0.6 M and an average marker density of 0.1 M. The significance thresholds for false positive results with α = 0.05 and α = 0.01 correspond to LRT values > 7.2 and LRT > 10.2, respectively.

### Structural analysis of the ACSL1 gene

The coding sequence of the bovine *ACSL1 *gene is represented by the reference mRNA sequence NM_001076085.1, which spans 3690 bp and is located on BTA27.

At the beginning of our study, the previous bovine genome assembly Btau4.0 and the current reference assembly Btau4.2 available at NCBI (http://blast.ncbi.nlm.nih.gov/Blast.cgi?PAGE_TYPE=BlastSearch&PROG_DEF=blastn&BLAST_PROG_DEF=megaBlast&SHOW_DEFAULTS=on&BLAST_SPEC=OGP__9913__10708, [62]) annotated the bovine *ACSL1 *gene with a total of 19 protein-coding exons. *In silico *sequence analysis of the respective mRNA and protein sequences (NM_001076085.1 and NP_001069553) revealed that parts of the sequences could not be aligned to the bovine genome reference assembly Btau4.2. This indicated an incomplete annotation of the bovine *ACSL1 *gene. However, in the alternative bovine genome assembly Bos_taurus_UMD3.1 (ftp://ftp.cbcb.umd.edu/pub/data/assembly/Bos_taurus/Bos_taurus_UMD_3.1/, [63]) integrated into the recent bovine genome assembly, Build 5.2, at NCBI (http://www.ncbi.nlm.nih.gov/projects/mapview/map_search.cgi?taxid=9913), the bovine *ACSL1 *gene was annotated with a total of 21 protein-coding exons, which is also in agreement with the earlier bovine genome assembly, version Btau3.1. Comparative sequence analysis between gene and protein sequences of the bovine *ACSL1 *gene and those of the orthologous human counterparts (NM_001995.2 and NP_001986.2) and the current human genome assembly Hsa37.2 (http://blast.ncbi.nlm.nih.gov/Blast.cgi?PAGE_TYPE=BlastSearch&PROG_DEF=blastn&BLAST_PROG_DEF=megaBlast&SHOW_DEFAULTS=on&SHOW_DEFAULTS=on&BLAST_SPEC=OGP__9606__9558) showed that the mRNA and amino acid sequences of both species display a high similarity (88% and 91% identity, respectively), which supported the annotation of the current Bos_taurus_UMD3.1 and the earlier Btau3.1 assemblies.

An experimental confirmation of the bovine *ACSL1 *gene structure was required because of the inconsistent annotation of the ACSL1 gene. Therefore, fragments completely covering the coding region of the gene and the 5' und 3' UTRs, including the respective critical gene fragments with discordant structure annotation, were validated in our study. Exon-flanking primers (Table 4) were derived from the sequence contigs NW_001494406.2 and NW_930554.1 and used for PCR-amplification with genomic DNA and cDNA. Genomic DNA was isolated from blood leucocytes using standard methods. The cDNA was prepared from liver tissue of a lactating cow. Total RNA extraction and cDNA synthesis by reverse transcription were performed as described recently [43]. To amplify cDNA fragments of the *ACSL1 *gene, PCR was performed with cDNA using gene-fragment specific primers (Table 4). The PCR-amplicons were isolated from agarose gels using the NucleoSpin^® ^Extract II kit (Macherey & Nagel) and sequenced with PCR primers using BigDye^© ^sequencing chemistry on a capillary sequencer (MEGABACE, GE Healthcare).

**Table 4:** Primer sequences for the bovine ACSL1 gene applied for annotation confirmation, screening for polymorphisms and genotyping

Primer	Sequence (5' → 3')	Gene region	Amplicon (bp)	Position in reference sequence	AccNo. of reference sequence	Application*
ACSL1_F1 ACSL1_R1	CCGAGCCCCAACCGAGAC TGGACGCTGTTCTTGAGTGGTG	intron 1 -promoter	844	1918181 - 1918198 1919003 - 1919024	NW_001494406.2	SNP
ACSL1_E1_F3 ACSL1_E1SF	GACCCGAGCCCCAACCGAG GTTGAGCCACCACAATTTACTC	intron 1 -promoter	497	1918178 - 1918196 1918674 - 1918653	NW_001494406.2	SNP
ACSL1_E1SR ACSL1_R1	GGACTGCCCTGGATTTCACAAG TGGACGCTGTTCTTGAGTGGTG	promoter	413	1918612 - 1918633 1919003 - 1919024	NW_001494406.2	SNP
ACSL1_F2 ACSL1_R2	TCGCTGCTGAAGTCCTGTCTG GCTCTAATGCCCCCGTTGATG	exon 2	501	1897290 - 1897310 1897770 - 1897790	NW_001494406.2	SNP
ACSL1_F3 ACSL1_R3	TTGCGTGGGAGAGAGTTGTG TCAGGTGGAGGATTTATGTCAG	exon 3	384	1882758 - 1882777 1883120 - 1883141	NW_001494406.2	SNP
ACSL1_F4 ACSL1_R4	GCATCCACACTCCATAGAAAC AATAAAGAAGCAAAACTCAGACC	exon 4	345	1881998 - 1882018 1882320 - 1882342	NW_001494406.2	SNP
ACSL1_F5 ACSL1_R5	ATGAAAGGGAAAAGTGAAAGTG CTTGAGTTGGACCTGATGCTG	exon 5	457	1878288 - 1878309 1878724 - 1878744	NW_001494406.2	SNP
ACSL1_F6 ACSL1_R6	CGGCTGGAAGTAAAGAGACAC TTGTGTTCTTCATCCTCCTTTC	exon 6	574	1875840 - 1875860 1876392 - 1876413	NW_001494406.2	SNP
ACSL1_F7 ACSL1_R7	GTTCTCTTTTACAGGACCAG CAGGGATGCTTTACTTACTC	exon 7	600	1875542 - 1875561 1876122 - 1876141	NW_001494406.2	SNP
ACSL1_F8_9 ACSL1_R8_9	TGGGTGATGTAAATGTGTGAGG ATGATAGGAATGGCAGTGGAGAC	exons 8-9	750	1872567 - 1872588 1873294 - 1873316	NW_001494406.2	SNP
ACSL1_F10 ACSL1_R10	ATCTGTATTTCAGGTACTGTTTC GTTTATGGGCTTCTCTCACG	exon 10	287	1871656 - 1871678 1871923 - 1871942	NW_001494406.2	SNP
ACSL1_F11 ACSL1_R11	TACACACTTGAACTTACCAG TGTGCTCTGAAATAAATGG	exon 11	314	1869171 - 1869190 1869466 - 1869484	NW_001494406.2	SNP
ACSL1_F12 ACSL1_R12	TCTGTATTGTGCCTTCTGATG GGAAACTGGGCTGAAATGC	exon 12	371	1866801 - 1866821 1867153 - 1867171	NW_001494406.2	SNP
ACSL1_F13 ACSL1_R13	TCTCACACAATAAAGGGGTAGG TCCACATCTTCACCAACACTC	exon 13	516	1864669 - 1864690 1865164 - 1865184	NW_001494406.2	SNP
ACSL1_F14 ACSL1_R14	AAGCCGCCCAGGAATAACAC TGCCACAAACCCACGACACT	exon 14	516	1863888 - 1863907 1864384 - 1864403	NW_001494406.2	SNP
ACSL1_F15 ACSL1_R15	GACTTGTGTTTATTTCTGCCTG TGGGCTGAGGTTTCTAATCC	exon 15	524	1862774 - 1862795 1863278 - 1863297	NW_001494406.2	SNP
ACSL1_F16 ACSL1_R16	TGCTGAGAAGTGGCTGGTTAC CATGAGAACAGGGCTTATTGG	exon 16	247	1860135 - 1860155 1860361 - 1860381	NW_001494406.2	SNP
ACSL1_F17 ACSL1_R17	ATGCGAGGGAGAAAGAGG CCGCTAACAAAAAGAACAGTG	exon 17	427	1859039 - 1859056 1859445 - 1859465	NW_001494406.2	SNP
ACSL1_F18 ACSL1_R18	GGCAAACTTCCCATTACACTG GACTCCTTCATCCCTTCTCTG	exon 18	512	1857386 - 1857406 1857877 - 1857897	NW_001494406.2	SNP
ACSL1_F19_20 ACSL1_R19_20	GCCAAAGCACACCACTCTC CGAAGCAGATAATAAGGAACTAC	exons 19-20	517	139425 - 139443 139919 - 139941	NW_930554.1	SNP
ACSL1_F21 ACSL1_R21	CACCCGCCTTTGTAACTG GTCCTGATTCTGTCCTGATGTC	exon 21	548	138819 - 138836 139345 - 139366	NW_930554.1	SNP
ACSL1_UTR_F31 ACSL1_UTR_R3	AAACCCTCTGGTCCTCTTGCG CAATGGCAGGAAGGGAGGGAG	exon 21	404	138633 - 138653 139016 - 139036	NW_930554.1	SNP
ACSL1_UTR_F2 ACSL1_UTR_R21	GAGTTTTCCAGATTCCTATGTCC CCTGTTACCCTCCCTTCCCTG	exon 21	650	137966 - 137988 138595 - 138615	NW_930554.1	SNP
ACSL1_UTR_F11 ACSL1_UTR_R1	ATGCGACTGCTGACATGAAAAAC AAATAAATGCTCTTCTGTCGTAATG	exon 21	527	137530 - 137552 138032 - 138056	NW_930554.1	SNP
						
ACSL1_E1_F3 ACSL1_E1_R2	GACCCGAGCCCCAACCGAG GCTCGTAGGCTGCAGCGAG	intron 1-promoter	220	1918178 - 1918196 1918379 - 1918397	NW_001494406.2	GT
ACSL1_F7 ACSL1_R7	GTTCTCTTTTACAGGACCAG CAGGGATGCTTTACTTACTC	exon 7	600	1875542 - 1875561 1876122 - 1876141	NW_001494406.2	GT (PCR-RFLP)
ACSL1_E1_F5 ACSL1_E1_R3 ACSL1_E1_F7_T ACSL1_E1_R8_C	GGAGGGAACTCGGGGAGCC AGGGCGGGGCTGAGACGG GCTATTTAAGGGTGCCGCCGT GCAGCCAGCTCTCGGAAGTAG	promoter	451 316 175	1918052 - 1918070 1918485 - 1918502 1918328 - 1918348 1918348 - 1918368	NW_001494406.2	GT (Tetra-ARMS PCR)
						
ACSL1_E1_F2 ACSL1_E5_R1	CGGAGGAGACTGTGGCTTAG CTGAGCGAAGATGCCAATAAAC	exons 1-5	505	38 - 58 521 - 543	NM_001076085.1	cDNA
ACSL1_E5_F1 ACSL1_E12_R2	CAGTTTATTGGCATCTTCGCTC GGAAGATGGTGGGTTGAAGG	exons 5-12	649	519 - 541 1148 - 1168	NM_001076085.1	cDNA
ACSL1_E11_F2 ACSL1_E18_R1	CCATATGTTTGAGAGAGTTGTAG ATGTACTCCCCCTGTGCCAG	exons 11-18	735	1046-1069 1761 - 1781	NM_001076085.1	cDNA
ACSL1_E17_F2 ACSL1_E21_R2	CTGGATAAAGACGGCTGGTTG GAGTTCAGGGTGGAGATAGATG	exons 17-21	399	1665 - 1686 2042 - 2064	NM_001076085.1	cDNA
ACSL1_E21_R3	GTCAAACTCCCCTCCGCTTC	exons 17-21	540	2185 - 2205	NM_001076085.1	cDNA, RT
ACSL1_E21_R4	CAGAAAGAGCAAAGTCCTAACC			2454 - 2476	NM_001076085.1	cDNA, RT

* cDNA: analysis of cDNA structure, RT: reverse transcription, GT: genotyping, SNP: screening for polymorphisms

### Screening for polymorphisms in the ACSL1 gene

Screening for polymorphisms was carried out by re-sequencing and covered the complete coding sequence, exon-flanking intronic regions, the 5' and 3' UTRs and 724 bp of the promoter of the *ACSL1 *gene. DNA primer pairs for PCR amplification and sequencing were designed based on genomic contig sequences (NW_001494406.2 and NW_930554.1) and the mRNA sequence (NM_001076085.1), respectively (Table 4).

Four genomic DNA pools consisting of selected animals from the Charolais × Holstein resource population differing in their intramuscular fat content and index of delta 9-desaturase were established and subjected to screening for gene variants by comparative re-sequencing. The IMF pools contained DNA from sampling time- and pedigree-matched animals with high (n = 5, 4.93 ± 1.73%) and low (n = 7, 1.78 ± 0.21%) IMF. The Δ^9 ^desaturase index pools consisted of DNA from sampling time- and pedigree-matched animals with a high (n = 7; 50.87 ± 0.89) or low (n = 6, 43.86 ± 0.95) Δ^9 ^desaturase index. Furthermore, two genomic DNA samples from control individuals and two individual DNA samples originating from extreme animals displaying the lowest (1.63%) and highest (6.09%) IMF were included to validate the results received from the pools.

Genomic DNA was isolated from blood leucocytes using standard methods. PCR with exon-flanking primers (Table 4) was performed with a total of 60 ng genomic DNA as described above. The generated PCR products were purified using the peqGOLD Cycle-Pure Kit (PEQLAB) according to the manufacturer's instructions and sequenced. Sequencing was performed on a capillary sequencer (MEGABACE, GE Healthcare) with primers used for targeted PCR amplification. To identify variable DNA positions, the sequences were analyzed meticulously by visual inspection of the sequencing profiles from DNA-pools and individuals' DNA and by sequence alignment to the reference cDNA sequence (NM_001076085.1) as well as to the respective bovine genome sequences. All SNPs identified by sequencing of DNA pools were verified by single sample re-sequencing.

### SNP Genotyping

Out of the identified 19 *ACSL1 *SNPs (see Table 3, Figure 1), nine were genotyped in the Charolais × German Holstein resource population: Two exonic SNPs (*c.516C>G, c.1938T>G*) and five intronic SNPs (*c.481-233A>G, c.580+114C>G, c.845-58T>G, c.1267-100C>T, c.1959+56G>A*) were genotyped on an Illumina Beadstation [64] as part of a targeted 384 SNP GoldenGate assay. The SNP in exon 7 (*c.584A>G*) was analyzed using a PCR-RFLP assay with primers for amplification of the targeted region (Table 4) and the restriction enzyme *SacI *(Fermentas). The promoter SNP *c.-122G>A *was genotyped by a Tetra-ARMS PCR assay [65] and validated by direct sequencing. The respective primers are given in Table 4. The *NCAPG I442M *mutation was genotyped by PCR-RFLP [43].

### Association analysis

Prior to association analysis, we tested whether the phenotypic data of the individual traits were normally distributed using the Shapiro Wilk test. For those data displaying distributions significantly different from normality (P < 0.01), we performed natural log (ln) transformation, and the log- transformed data were subjected to association analysis. The respective data are indicated in Tables 2 and 3.

The BTA27 marker haplotypes of the individuals of the resource population were estimated by a Markov chain Monte Carlo (MCMC) algorithm implemented in Qxpak [60]. The corresponding haplotypes were submitted to pairwise LD analysis calculating r^2 ^values using PowerMarker V3.25 [66].

Subsequently to the QTL analyses, association analyses were performed between ACSL1 gene SNPs and the absolute and relative FA composition traits in *M. longissimus dorsi*. The following model testing for LD as implemented in Qxpak [60] was applied:

$$y_{i}=a_{p}+\underset{k}{\sum }\underset{h}{\sum }\lambda_{ikh}g_{k}+\underset{m}{\sum }\underset{n}{\sum }\lambda_{imn}g_{m}+u_{i}+e_{ihkmnp}$$

where y_i _is the record of individual i, a_p _is the fixed effect of slaughter year p, λ_ihk _is an indicator variable for the *NCAPG I442M *locus, which is 1 when the allele at the h^th ^haplotype (1 or 2) of the i^th ^individual is 1 and otherwise 0, λ_imn _is a respective indicator variable for the specific *ACSL1 *SNP, u_i _is the infinitesimal genetic effect of individual i, g_k _and g_m _are the respective allelic effects for *NCAPG I442M *and the *ACSL1 *SNP, and e_ihkmnp _is the residual. Analogous to the QTL analyses, the *NCAPG I442M *mutation was included in the model, because previous analyses had shown a major effect of the mutation on carcass lipid deposition and growth in the resource population [44]. A likelihood-ratio test (likelihood of model with both loci vs. likelihood of model with *NCAPG I442M*) was applied to test for statistical significance. In order to dissect whether the association of the respective *ACSL1 *variant with intramuscular FA composition is solely due to indirect effects on IMF or a consequence of direct effects on the specific FA accumulation, we extended the model and fitted IMF as an additional covariate. A Bonferroni correction was calculated (q-value) to account for testing several SNPs in order to avoid false positive associations. The q-values thresholds of 0.05 and 0.1, respectively, indicate an experiment-wise significant or suggestive association, respectively. Finally, an additive fixed effect of the SNP in intron 5 was added in the QTL model described above to test whether this SNP might explain the QTL variance at the identified position on BTA27.

## Abbreviations

(*SCD1)*: stearoyl-Coenzyme A desaturase 1; (*SREBP-1)*: sterol regulatory element binding protein 1; (*FASN)*: fatty acid synthase; (*FABP4)*: fatty acid binding protein 4; *(LXRα)*: liver X receptor alpha; (*GH)*: growth hormone; *(ACACA)*: acetyl-CoA carboxylase alpha; (*NCAPG)*: non-SMC condensin I complex; subunit G, (*MSTN*): myostatin.

## Authors' contributions

PW participated in screening for polymorphisms, genotyping and statistical analysis. KN carried out fatty acid analysis. CK conceived the study and performed statistical analysis. RW performed gene structure analysis and screening for polymorphisms. CK and RW wrote the manuscript. All authors read and approved the final manuscript.
